# A novel human S10F‐Hsp20 mutation induces lethal peripartum cardiomyopathy

**DOI:** 10.1111/jcmm.13665

**Published:** 2018-05-15

**Authors:** Guan‐Sheng Liu, George Gardner, George Adly, Min Jiang, Wen‐Feng Cai, Chi Keung Lam, Fawzi Alogaili, Nathan Robbins, Jack Rubinstein, Evangelia G. Kranias

**Affiliations:** ^1^ Department of Pharmacology & Systems Physiology University of Cincinnati College of Medicine Cincinnati OH USA; ^2^ Department of Internal Medicine University of Cincinnati College of Medicine Cincinnati OH USA; ^3^ Department of Pathology & Lab Medicine University of Cincinnati College of Medicine Cincinnati OH USA; ^4^ Molecular Biology Division Center for Basic Research Biomedical Research Foundation of the Academy of Athens Athens Greece

**Keywords:** apoptosis, Hsp20, human mutation, pathological hypertrophy, peripartum cardiomyopathy

## Abstract

Heat shock protein 20 (Hsp20) has been shown to be a critical regulator of cardiomyocyte survival upon cardiac stress. In this study, we investigated the functional significance of a novel human Hsp20 mutation (S10F) in peripartum cardiomyopathy. Previous findings showed that cardiac‐specific overexpression of this mutant were associated with reduced autophagy, left ventricular dysfunction and early death in male mice. However, this study indicates that females have normal function with no alterations in autophagy but died within a week after 1‐4 pregnancies. Further examination of mutant females revealed left ventricular chamber dilation and hypertrophic remodelling. Echocardiography demonstrated increases in left ventricular end‐systolic volume and left ventricular end‐diastolic volume, while ejection fraction and fractional shortening were depressed following pregnancy. Subsequent studies revealed that cardiomyocyte apoptosis was elevated in mutant female hearts after the third delivery, associated with decreases in the levels of Bcl‐2/Bax and Akt phosphorylation. These results indicate that the human S10F mutant is associated with dysregulation of cell survival signalling, accelerated heart failure and early death post‐partum.

## 1 INTRODUCTION

Heat shock protein 20 (Hsp20) is one of the 10 members of the small heat shock protein family, which are upregulated under stress conditions and have been suggested to play a critical role in cell survival.^1^ Hsp20 is ubiquitously expressed and is highly abundant in cardiac, skeletal and smooth muscles.^1^ The levels of Hsp20 are increased in cardiac ischemia/reperfusion (I/R) injury and heart failure,^2^ which may represent a potential cardioprotective mechanism. Indeed, cardiac overexpression of Hsp20 elicited protection against apoptosis at both the cellular and whole heart levels.^3^, ^4^, ^5^, ^6^ The underlying mechanisms appeared to include inhibition of Ask1^3^ and preservation of Akt activity^4^ as well as Bcl‐2/Bax levels.^5^ Accordingly, reduction in the Hsp20 levels, by overexpression of miRNA‐320, resulted in substantial cardiomyocyte loss and increased infarct size following I/R.^7^ Collectively, these studies indicate that Hsp20 is a critical mediator of protection against apoptosis and functional decreases of this protein may result in increased susceptibility of the heart to cardiac stress.

We have recently identified a human mutation in the Hsp20 gene, which results in an amino acid change from serine to phenylalanine at position 10 (S10F).^8^ Transgenic (TG) male mice with cardiac overexpression of this mutation presented with impaired autophagy and increased apoptosis at an early age, resulting in cardiac dysfunction, heart failure and reduced survival.^8^ These findings prompted us to examine the role of the human S10F‐Hsp20 mutation in females and specifically its effects on cardiac function and remodelling upon pregnancy. Previous studies have implicated apoptosis as a contributing factor to the pathogenesis of peripartum cardiomyopathy (PPCM),^9^, ^10^, ^11^, ^12^, ^13^ associated with left ventricular systolic dysfunction, which develops in the last month of pregnancy or 5 months following delivery.^14^, ^15^, ^16^ Indeed, a study by Hayakawa et al^10^ demonstrated that inhibition of apoptosis via a caspase inhibitor improved left ventricular function and survival in a PPCM genetic mouse model. In support of this notion, deletion of the pro‐apoptotic protein Nix/Bnip3L reduced apoptosis and ventricular remodelling and enhanced cardiac function and survival, further highlighting the significance of apoptosis in the pathophysiology of PPCM.^12^, ^13^

In this study, the human S10F mutation resulted in progressively reduced survival in TG females after pregnancy, while there were no parallel deaths in non‐transgenic (NTG) females. Following the third pregnancy, the mutant females displayed significant ventricular remodelling, reduced cardiac function and increased apoptosis associated with reduced Bcl‐2/Bax and p‐AKT. These results suggest that the presence of the human S10F mutation may compromise the heart's ability to cope with pregnancy.

## 2 MATERIALS AND METHODS

### 2.1 Animal experiments

S10F‐Hsp20 TG mice^8^ were used in this study and their NTG littermates served as controls. Female mice were first bred at 12 weeks of age and the effects of pregnancy on survival were examined in S10F females (n = 19) and NTG females (n = 24). Separate cohorts of NTG and S10F females were bred for functional and cellular studies, performed after the third delivery. Echocardiographic assessment of left ventricular function and dimensions was performed within 3 days of delivery. Immediately after echocardiography, mouse hearts were excised following anaesthesia with sodium pentobarbital (70 mg/kg, ip) and processed for biochemical and cellular studies. Age‐matched non‐pregnant females (TG and NTG) were studied in parallel. All animal procedures were performed according to the NIH guidelines (Guide for the care and use of laboratory animals) and the Institutional Animal Care and Use Committee at the University of Cincinnati.

### 2.2 Western blot analysis

Hearts were snap frozen in liquid nitrogen and homogenized in cell lysis buffer (Sigma‐Aldrich) containing protease inhibitor cocktail (Roche) and phosphatase inhibitor cocktail (Bimake). Proteins were separated on an SDS gel and transferred to a nitrocellulose membrane. Following transfer, membranes were blocked with Licor Blocking Buffer for 1 hour and then probed with the corresponding antibody diluted 1:1000 in blocking buffer overnight at 4°C (Beclin1, Bcl‐2, Bax, p‐Akt and Akt were from Cell Signaling Technology; Hsp20 was from Fitzgerald; and GAPDH was from Santa Cruz Biotechnology). This step was followed by incubation with Licor secondary antibody (IRDye 680RD; IRDye800CW) at a dilution of 1:5000 and visualization using the Licor Odyssey Imager. Protein band intensity was quantified using AlphaEaseFC Software.

### 2.3 Evaluation of apoptosis

Terminal deoxynucleotidyl transferase dUTP nick end labelling (TUNEL) assay was performed, as previously described.^2^, ^3^ Briefly, hearts were rapidly extracted and fixed in 10% formalin (Sigma, St. Louis, MO, USA). Ventricular tissue was paraffin embedded and sectioned (5 μm), and was performed by the Department of Pathology at the Cincinnati Children Hospital Medical Center. Apoptotic nuclei were determined using the DeadEnd™ Fluorometric TUNEL system (Promega, Madison, WI, USA), according to the manufacturer's instruction. Sections were stained with Actc1 (Sigma‐Aldrich; 1:50) for cardiomyocyte visualization and mounted with VECTASHIELD^®^ Antifade Mounting Medium Plus DAPI to stain nuclei. TUNEL‐positive nuclei were examined with a fluorescence microscope.^2^, ^3^ Caspase‐3 activity was determined in NTG and S10F cardiac homogenates, using the Caspase‐3 Colorimetric Assay Kit (BioVision Incorporated, K105), in accordance with the manufacturer's instruction.

### 2.4 Echocardiography

Echocardiographic analysis was performed with a Vevo 2100 Ultrasound system (Visualsonics, Toronto, Canada), as previously described.^17^, ^18^ Briefly, mice were anaesthetized with isoflurane (1.5%‐2%), and images were obtained from a parasternal long axis view between 2 and 10 mm in depth in both M‐mode and B‐mode. Images were analysed in a blinded manner using Vevostrain software (Vevo 2100, v1.1.1 B1455, Visualsonic, Toronto, Canada).^17^, ^18^

### 2.5 Morphological analysis

Hearts were removed from the chest cavity and weighed on a Mettler Toledo, AT201 analytical scale. The skeletal muscle of the lower hindlimbs was dissected out, and the length of the left tibia was measured. Heart sections, as described above, were stained with haematoxylin (Sigma‐Aldrich, H9627) and eosin (Sigma‐Aldrich, E4009), and was carried out by the Department of Pathology at the Cincinnati Children's Hospital Medical Center. Sections were examined for sarcolemma staining labelled with wheat germ agglutinin (WGA, Invitrogen), according to manufacturer's instruction. Cardiomyocyte cross‐sectional area was measured using ImageJ Software.^4^, ^8^

### 2.6 Quantitative real‐time PCR assay

Total RNA was extracted and purified from heart tissue with a miRNeasy Mini Kit (QIAGEN). The first‐strand cDNA were generated from total RNA (1 μg) with a reverse transcriptase kit (Invitrogen). PCR was performed with a Bio‐Rad real‐time thermal cycler by using the following specific primer sequences: mouse ANP (Forward) 5′‐GAGAAGATGCCGGTAGAAGA‐3′, (Reverse) 5′‐AAGCACTGCCGTCTCTCAGA‐3′; mouse BNP (Forward) 5′‐AGGGAGAACACGGCATCATT‐3′, (Reverse) 5′‐GACAGCACCTTCAGGAGAT‐3′; and mouse GAPDH (Forward) 5′‐TCAACAGCAACTCCCACTCTT‐3′, (Reverse) 5′‐ACCCTGTTGCTGTAGCCGTATTCA‐3′. Relative expression of mRNA was calculated using the comparative threshold cycle (Ct) method, and values were normalized to GAPDH.^19^

### 2.7 Statistical analysis

Data were expressed as the mean ± SEM. Comparisons between two groups were performed with unpaired Student's *t* test. Comparison of results from more than two groups was analysed using ANOVA followed by the Holm‐Sidak post‐test. Analysis of survival was performed with the Kaplan‐Meier method. Results were considered statistically significant at *P* < .05.

## 3 RESULTS

### 3.1 Human S10F‐Hsp20 does not alter cardiac function or autophagy in female mice

**Figure 1:**
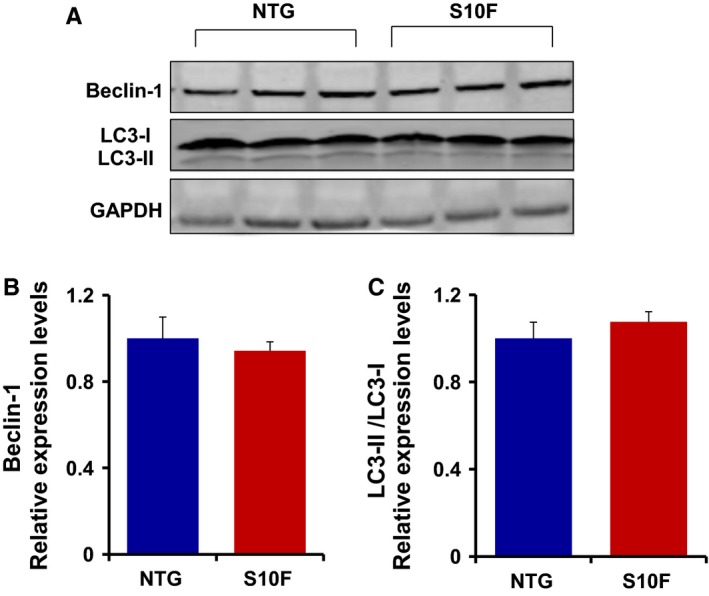
Autophagy is not altered in S10F‐Hsp20 females. (A) Representative western blots illustrating Beclin‐1 and LC3‐II/LC3‐I protein expression levels in S10F and non‐transgenic (NTG) female hearts at 6 months of age, with GAPDH as a loading control. Quantitative analysis of: (B) Beclin‐1 and (C) LC3‐II/LC3‐I protein expression levels in S10F and NTG hearts after normalization to GAPDH. Values represent mean ± SEM; n = 3 hearts per group

Assessment of cardiac function (EF: ejection fraction and FS: fractional shortening) and left ventricular chamber size (LVEDV: left ventricular end‐diastolic volume; LVESV: left ventricular end‐systolic volume; LVIDd: left ventricular internal dimension at end‐diastole; LVIDs: left ventricular internal dimension at end‐systole) by echocardiography indicated no differences in S10F females compared to NTGs up to 6 months of age (Table S1). In addition, we detected no changes in Beclin‐1 or LC3‐II/LC3‐1 expression in S10F females (Figure 1A‐C), suggesting that autophagy was preserved in these hearts. These results highlight a unique gender difference in the S10F transgenic model.

### 3.2 Increased deaths in S10F‐Hsp20 females after multiple pregnancies

**Figure 2:**
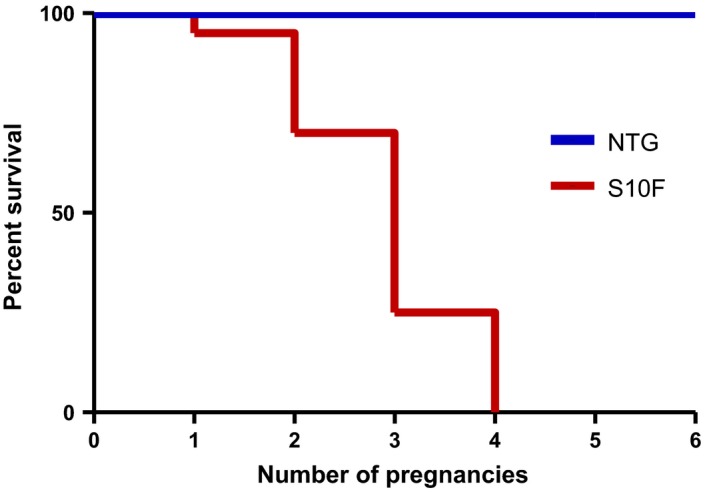
Reduced post partum survival of S10F‐Hsp20 females. (A) Kaplan‐Meier survival curve in female pregnant S10F vs non‐transgenic (NTG) mice; n = 24 for NTG and n = 19 for S10F. The deaths in the S10F‐Hsp20 group include: one after one pregnancy, four after two pregnancies, nine after three pregnancies and five after four pregnancies

Although mutant females showed no pathological changes compared to mutant males under basal conditions, they exhibited reduced survival upon pregnancy. Female TGs were fertile and delivered normal litter sizes, but they invariably died after 1‐4 pregnancies (Figure 2). Approximately 5% of the mutant females died after one pregnancy, 20% after 2 pregnancies, 70% after 3 pregnancies and all of them (n = 19) died after 4 pregnancies (Figure 2). In comparison, none of the NTG females (n = 24) died even after 6 pregnancies (Figure 2).

### 3.3 Increased cardiac dilation and reduced function in S10F‐Hsp20 females post‐partum

**Figure 3:**
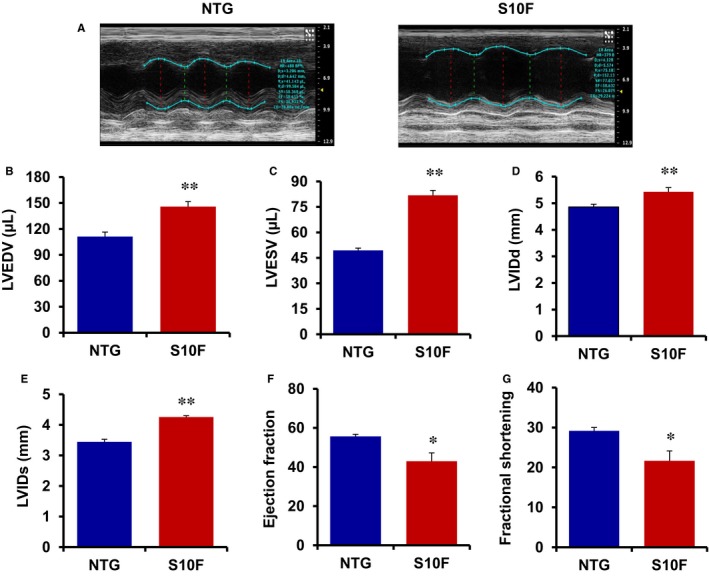
Increased left ventricular dilation and depressed cardiac function in post‐partum S10F‐Hsp20 females. (A) Sample M‐mode echocardiograms of female S10F and non‐transgenic (NTG) hearts following 3 pregnancies. Analysis of (B) left ventricular end‐diastolic volume (LVEDV); (C) left ventricular end‐systolic volume (LVESV); (D) LVIDd; (E) left ventricular internal dimension at end‐diastole (LVIDs); (F) ejection fraction (EF) %; and (G) fractional shortening (FS) % in mice of the indicated groups. Values represent mean ± SEM; n = 5 for NTG and n = 7 for S10F. *: *P* < .05 vs NTG; **: *P* < .01 vs NTG

Physiological pregnancy has been shown to alter cardiac function with significant increases in both heart rate and cardiac output.^20^ To assess whether the detrimental effects of mutant‐Hsp20 on survival (Figure 2) were associated with alterations in cardiac function or structure, we performed echocardiography on the NTG and mutant mice after 3 pregnancies. Although basal contractile and geometric parameters of mutant hearts were similar to NTGs up to 6 months of age (Table S1), there was significant cardiac dilation after 3 pregnancies (Figure 3A‐E). Specifically, left ventricular chamber volume (LVEDV and LVESV) and internal dimensions (LVIDd and LVIDs) were significantly increased in mutant mice, compared to NTGs (Figure 3B‐E). Moreover, both EF and FS in mutant hearts were reduced to 75% of the values in NTGs (Figure 3F,G). Notably, EF and FS were not altered upon pregnancy in NTG females compared to their non‐pregnant counter‐parts (Figure 3F,G compared to Table S1). In addition, there was a significant decrease in heart rate of mutant mice compared to NTGs post‐partum (Table S2). The echocardiographic tracings were reviewed and all mice were in normal sinus rhythm with normal atrioventricular conduction. There were no significant atrial or ventricular arrhythmias noted in any of the mice. Overall, these data suggest that the S10F‐Hsp20 mutation significantly compromises cardiac function and leads to left ventricular cavity dilation upon pregnancy in female mice.

### 3.4 Increased cardiac remodelling in S10F‐Hsp20 female mice

**Figure 4:**
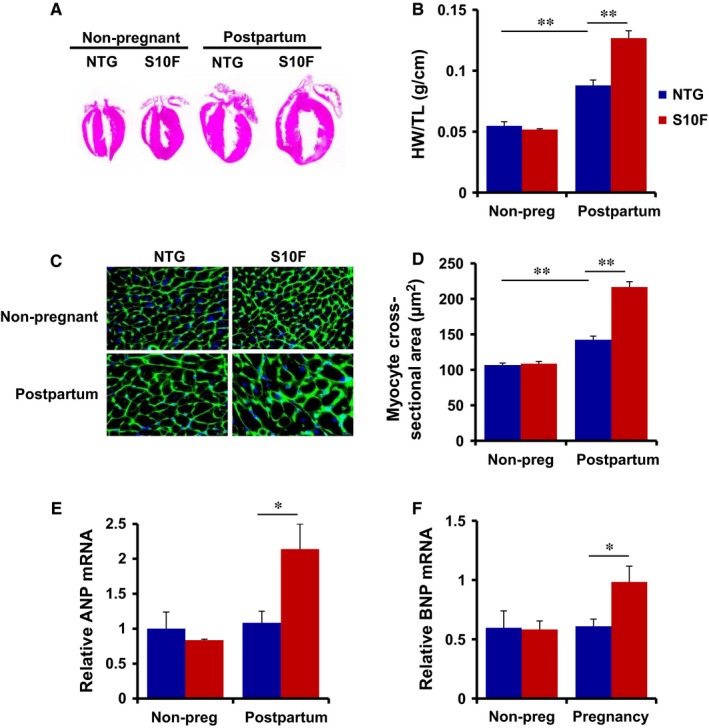
Cardiac remodelling in post‐partum S10F‐Hsp20 females. Studies were performed in non‐transgenic (NTG) and S10F females following 3 pregnancies with age‐matched non‐pregnant females as controls. (A) Haematoxylin staining of ventricular sections. (B) Ratio of heart weight (g)/tibia length (cm) (n = 4‐5 hearts/group); (C) immunostaining of ventricular sections with fluorescence labelled wheat germ agglutinin; and (D) quantitative analysis of cardiomyocyte cross‐sectional area (n = 5 hearts/group). mRNA expression levels of (E) atrial natriuretic peptide (ANP) and (F) brain natriuretic peptide (BNP) after normalization to GAPDH (n = 3 hearts/group). Values represent mean ± SEM. *: *P* < .05; ** *P* < .01

The alterations in cardiac dimensions, observed in S10F females following pregnancy, prompted us to determine whether hypertrophic remodelling was evident at the cellular and whole organ levels.^21^, ^22^, ^23^ Thus, we examined cardiac weight (HW) and analysed the ratio of heart weight over tibia length (TL) in NTG and mutant females after 3 pregnancies, with the age‐matched non‐pregnant mice as controls. There was no alteration in HW/TL ratio between mutant and NTG mice under non‐pregnancy conditions (Figure 4A,B). However, pregnancy resulted in increases of the HW/TL ratio by 1.6‐fold and 2.4‐fold in NTG and mutant mice respectively (Figure 4B). Accordingly, the cardiomyocyte cross‐sectional area in pregnant mice increased by 1.4‐fold in NTG and by 2.1‐fold in mutant hearts (Figure 4C,D). Furthermore, the mRNA levels of foetal gene hypertrophic markers, including atrial natriuretic peptide (ANP) and brain natriuretic peptide (BNP), were significantly upregulated in mutant post‐partum hearts compared to their non‐pregnant controls, while NTGs showed no differences (Figure 4E,F), indicating the development of pathological hypertrophy in the S10F hearts.

### 3.5 Increased cardiomyocyte apoptosis in S10F‐Hsp20 female hearts

**Figure 5:**
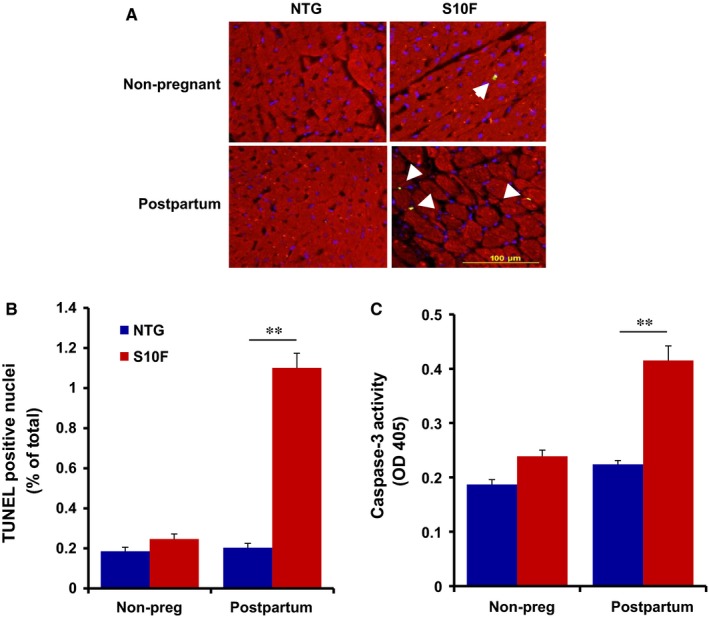
Elevated apoptosis in post‐partum S10F‐Hsp20 hearts. TUNEL staining, TUNEL‐positive nuclei and caspace‐3 activity in S10F and non‐transgenic (NTG) females after three pregnancies with age‐matched, non‐pregnant mice as controls. A, Representative images of TUNEL staining in heart sections (arrows indicate TUNEL‐positive nuclei). B, Quantitative analysis of the percentage of TUNEL‐positive nuclei relative to total nuclei of the indicated groups (n = 5 hearts/group). C, Caspase‐3 activity in heart homogenates (n = 3 hearts/group). Values represent mean ± SEM; **: *P* < .01

Accumulating evidence suggests that slight elevation of cardiomyocyte apoptosis can cause depressed cardiac contractility and heart failure upon pregnancy.^9^, ^10^, ^24^ To investigate the effects of the S10F‐Hsp20 human mutation on apoptosis, cardiomyocyte survival in female mutant hearts was assessed under non‐pregnant and post‐partum conditions, using TUNEL staining, with NTGs as controls. We observed no significant increase in apoptosis in mutant female hearts under non‐pregnant conditions, while the number of cardiomyocytes undergoing apoptosis increased by 5‐fold compared to NTGs, post‐partum (Figure 5A,B). To further confirm the increased apoptotic events, caspase‐3 activity was examined in heart homogenates. There was no significant alteration under non‐pregnant conditions, but caspase‐3 activity was approximately 2‐fold higher in mutant hearts compared to the NTGs, after 3 pregnancies (Figure 5C). These results demonstrate that the human S10F mutation is associated with significant increases in apoptosis upon pregnancy, resulting in cardiomyocyte loss, ventricular remodelling and cardiac dysfunction.

### 3.6 Apoptosis in S10F‐Hsp20 hearts is associated with decreases in Bcl2/Bax levels and Akt activity

**Figure 6:**
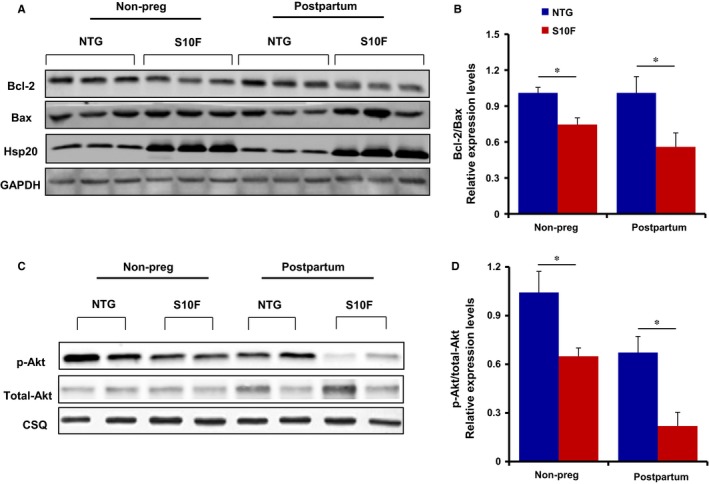
Reduced Bcl‐2/Bax and p‐Akt expression levels in post‐partum S10F‐Hsp20 female hearts. A, Representative western blots of Bcl‐2 and Bax protein expression levels in post‐partum S10F and non‐transgenic (NTG) hearts and age‐matched non‐pregnant controls, with GAPDH as the loading control. B, Quantitative analysis of the ratio of Bcl‐2/Bax expression levels after normalization to GAPDH. C, Representative western blots of p‐Akt and total‐Akt protein expression levels, with calsequestrin (CSQ) as the loading control. D, Quantitative analysis of the ratio of p‐Akt/total‐Akt after normalization to CSQ. Values represent mean ± SEM; n = 3 hearts per group. *: *P* < .05

To elucidate the potential mechanisms underlying increased cardiomyocyte loss in pregnant mutant hearts, we assessed the protein levels of Bcl‐2 (anti‐apoptotic) and Bax (pro‐apoptotic), as alterations in these proteins have been reported in cardiomyocytes undergoing apoptosis.^25^, ^26^ It was found that under post‐partum conditions the protein level of Bax was increased, whereas the level of Bcl‐2 was reduced in S10F hearts, compared to NTG controls (Figure S1). Hence, the relative ratio of Bcl‐2 to Bax was reduced by 40% in post‐partum mutant hearts compared to NTG females (Figure 6A,B). This ratio was lower in mutant hearts even in the absence of pregnancy but it decreased further following pregnancy. Additionally, Akt signalling, which is critical for cell survival,^27^, ^28^ was significantly diminished in post‐partum mutant hearts compared to NTGs, as demonstrated by reduced levels of p‐Akt (Figure 6C,D). It is worth noting that p‐Akt was lower in mutant hearts even in the absence of pregnancy (Figure 6C,D). The decreases of these protective pathways may contribute to the diminished ability of mutant female hearts to tolerate pregnancy. Interestingly, we detected no changes in Beclin‐1 or LC3‐II/LC3‐1 levels even after pregnancy (Figure S2), excluding the process of autophagy as a mediator of the increased apoptosis in the female hearts.

## 4 DISCUSSION

This study presents the first evidence that the presence of a human S10F mutation in the Hsp20 gene may compromise the heart's ability to cope with pregnancy, resulting in increased cardiomyocyte apoptosis, pathological remodelling and the development of peripartum cardiomyopathy. The pro‐survival effects of Hsp20^2^, ^3^, ^4^, ^5^, ^6^, ^7^ were abrogated in mutant hearts upon pregnancy, demonstrated by reduced Bcl‐2/Bax levels as well as Akt signalling. These findings suggest that Ser10 is crucial for the protective effects of Hsp20 and conversion of this amino acid to Phe contributes to the development of cardiomyopathy following pregnancy.

We recently reported that male mice with cardiac‐specific overexpression of the S10F‐Hsp20 mutant exhibited cardiac dysfunction by 6 months of age and early lethality (50% dead by 15 months).^8^ Upon further investigation, it was discovered that autophagy was significantly depressed in these hearts by 2 months of age because of reduced interaction between Hsp20 and Beclin‐1 and the resultant proteasomal degradation of Beclin‐1. This lack of autophagy led to substantial cardiomyocyte loss and ultimately heart failure in mutant male mice upon ageing.^8^ Conversely, the present study highlights a unique gender difference in the S10F‐Hsp20 model, with female hearts exhibiting no changes in Beclin‐1 levels even following multiple pregnancies. Although basal apoptosis was slightly elevated in mutant female hearts, these changes were minimal in comparison to males,^8^ and there were no alterations in cardiac function or survival in mutant females. However, when subjected to pregnancy, these females exhibited depressed cardiac function, pathological remodelling and early death.

Peripartum cardiomyopathy (PPCM) has been shown to be a life‐threatening disorder affecting approximately 1000‐1300 women in the United States per year, with much higher rates in developing nations.^14^, ^16^, ^29^ Although the aetiology underlying the pathogenesis of this condition is currently unclear, various mechanisms have been proposed. Growing evidence demonstrates that apoptosis, or programmed cell death, is a major contributing factor to the development of PPCM.^9^, ^10^, ^11^, ^12^, ^13^ Specifically, transgenic mice overexpressing Gαq developed lethal PPCM, associated with significant cardiomyocyte loss and ventricular fibrosis.^9^ Remarkably, pregnant Gαq transgenic mice infused with the caspase inhibitor IDN‐1965 exhibited enhanced cardiac function and complete rescue of mortality.^10^ These effects were associated with inhibition of cardiomyocyte apoptosis, highlighting the importance of this process in the development of PPCM. In a separate study, ablation of Nix/Bnip3L, a pro‐apoptotic protein which is transcriptionally upregulated in the Gαq model and mediates cytochrome‐c release, enhanced cardiomyocyte survival and function and prevented lethality.^11^, ^12^, ^13^ Thus, increases in cell death during pregnancy are critical for the development of heart failure post‐partum.

The Hsp20 protein has been shown to protect the heart against apoptosis through various mechanisms in response to stress.^2^, ^3^, ^4^, ^5^, ^6^ Transgenic mice with cardiac overexpression of Hsp20 exhibited full functional recovery and reduction in infarct size upon ischemia/reperfusion injury, associated with diminished caspase‐3‐induced apoptosis.^5^ Inhibition of cell death was associated with maintenance of the levels of the anti‐apoptotic Bcl‐2 and the pro‐apoptotic Bax in transgenic hearts, while NTGs demonstrated a reduced Bcl‐2/Bax ratio. In addition, apoptotic signalling promotes the translocation of Bax to mitochondria from the cytosol, inducing caspase‐mediated apoptosis. Notably, Hsp20 was shown to interact with Bax, preventing its translocation, thereby inhibiting cytochrome‐c release.^5^ In this study, female mutant hearts exhibited substantial cardiomyocyte loss upon the stress of pregnancy, associated with elevated caspase‐3 activity. At the molecular level, transgenic hearts showed a significant reduction in the levels of Bcl‐2/Bax, which may be a major contributing factor to the pro‐apoptotic effects and the development of PPCM. It is important to note that in contrast to males, which exhibited cardiomyocyte loss as a result of diminished autophagy,^8^ S10F females showed preservation of autophagy, indicating a separate mechanism is underlying the apoptosis in these hearts.

Heat shock protein 20 has also been implicated in the regulation of Akt signalling through direct interaction and stabilization of its phosphorylated state (p‐Akt). Specifically, cardiac overexpression of Hsp20 protected the heart against doxorubicin‐induced apoptosis and prolonged survival through preservation of Akt activity and inhibition of caspase‐3 cleavage.^4^ Previous studies have revealed the importance of Akt signalling in physiological hypertrophy and cell survival.^27^, ^28^, ^30^, ^31^, ^32^, ^33^ During pregnancy, the heart undergoes a series of physiological hypertrophic adaptations to cope with volume overload, which are reversible following pregnancy, similar to exercise.^23^, ^30^, ^34^, ^35^ At the molecular and cellular levels, the size of the cardiomyocyte increases in the absence of foetal gene (ANP, BNP) activation.^23^, ^30^ Indeed, at the whole heart level, wall thickness is increased, concomitant with proportional increases in ventricular chamber size.^22^, ^35^ These effects are consistent with our results in NTG pregnant mice, which exhibited physiological hypertrophy upon pregnancy, as demonstrated by proportional increases in heart weight/tibia length and chamber size without changes in foetal gene expression. In contrast, the mutant hearts exhibited extensive increases in chamber size as well as cardiomyocyte cross‐sectional area and heart weight/tibia length. At the molecular level, increases in foetal gene markers, ANP and BNP, indicated the development of pathological hypertrophy. Along these lines, Akt activity was depressed in mutant female hearts even under baseline conditions, compared to NTGs. Thus, diminished Akt signalling may predispose the mutant hearts to maladaptive, pathological hypertrophy in addition to reduced cell survival upon the stress of pregnancy. Interestingly, NTG hearts also showed a reduction in p‐Akt post‐partum which is consistent with previous studies, and although not entirely clear, may be because of hormonal changes and unloading of the heart after delivery.^31^, ^33^ Although the current findings underscore the importance of human S10F‐Hsp20 mutation in the development of PPCM, a limitation of the current work is the use of an overexpression strategy to examine the functional role of the mutant. A gene‐targeted knock‐in approach would have provided a more elegant setting to examine the S10F significance in vivo. However, the levels of Hsp20 increase about 10‐fold in heart failure,^2^ and this is one of the reasons that a transgenic model with cardiac overexpression of the human mutant to similar levels as those present in failing hearts was chosen for this study.

In summary, our findings indicate that the human S10F‐Hsp20 mutation compromises the heart's ability to cope with pregnancy, leading to extensive cardiomyocyte loss, depressed cardiac function, pathological remodelling and increased mortality. Indeed, this amino acid substitution diminishes regulation of Bcl‐2/Bax and Akt signalling, associated with increased apoptosis and pathological hypertrophy. These results suggest that S10F‐Hsp20 females may be more susceptible to the development of cardiomyopathy upon pregnancy. Future studies may be designed to further delineate the susceptibility of human S10F‐Hsp20 carriers to peripartum cardiomyopathy.

## CONFLICT OF INTEREST

The authors confirm that there are no conflicts of interest.

## AUTHOR CONTRIBUTIONS

GL and GG performed the research study and analysed the data; GG, GL and EGK wrote the paper; GL, CL and EGK designed the research study; GA, WC and FA performed some research; MJ, NR and JR performed the echocardiography and data analysis.
