# Supplementary material for: A novel human S10F‐Hsp20 mutation induces lethal peripartum cardiomyopathy
Source: J Cell Mol Med. 2018 May 15;22(8):3911–9. doi: 10.1111/jcmm.13665 (PMC6050507; doi:10.1111/jcmm.13665)
Supplement: Supplementary file 1 [file JCMM-22-3911-s001.pdf]

Figure S1

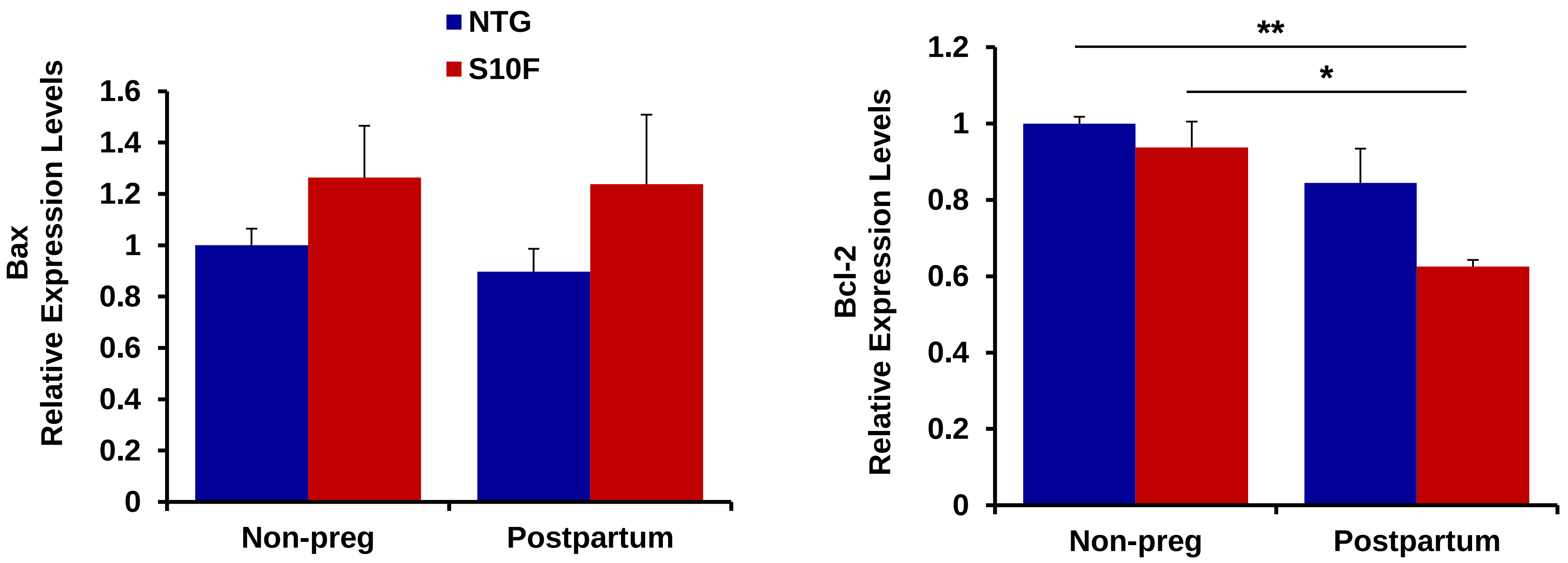

Supplementary Figure 1. Protein levels of Bax and Bcl-2 in non-pregnant and postpartum NTG and S10F-Hsp20 hearts. (A) Quantitative analysis of Bax and Bcl-2 protein levels after normalization to the loading control, GAPDH. Values represent mean ± SEM; n=3 per group. *: $p<0.05$; **: $p < 0.01$.
