# Supplementary material for: A novel human S10F‐Hsp20 mutation induces lethal peripartum cardiomyopathy
Source: J Cell Mol Med. 2018 May 15;22(8):3911–9. doi: 10.1111/jcmm.13665 (PMC6050507; doi:10.1111/jcmm.13665)
Supplement: Supplementary file 2 [file JCMM-22-3911-s002.pdf]

Figure S2

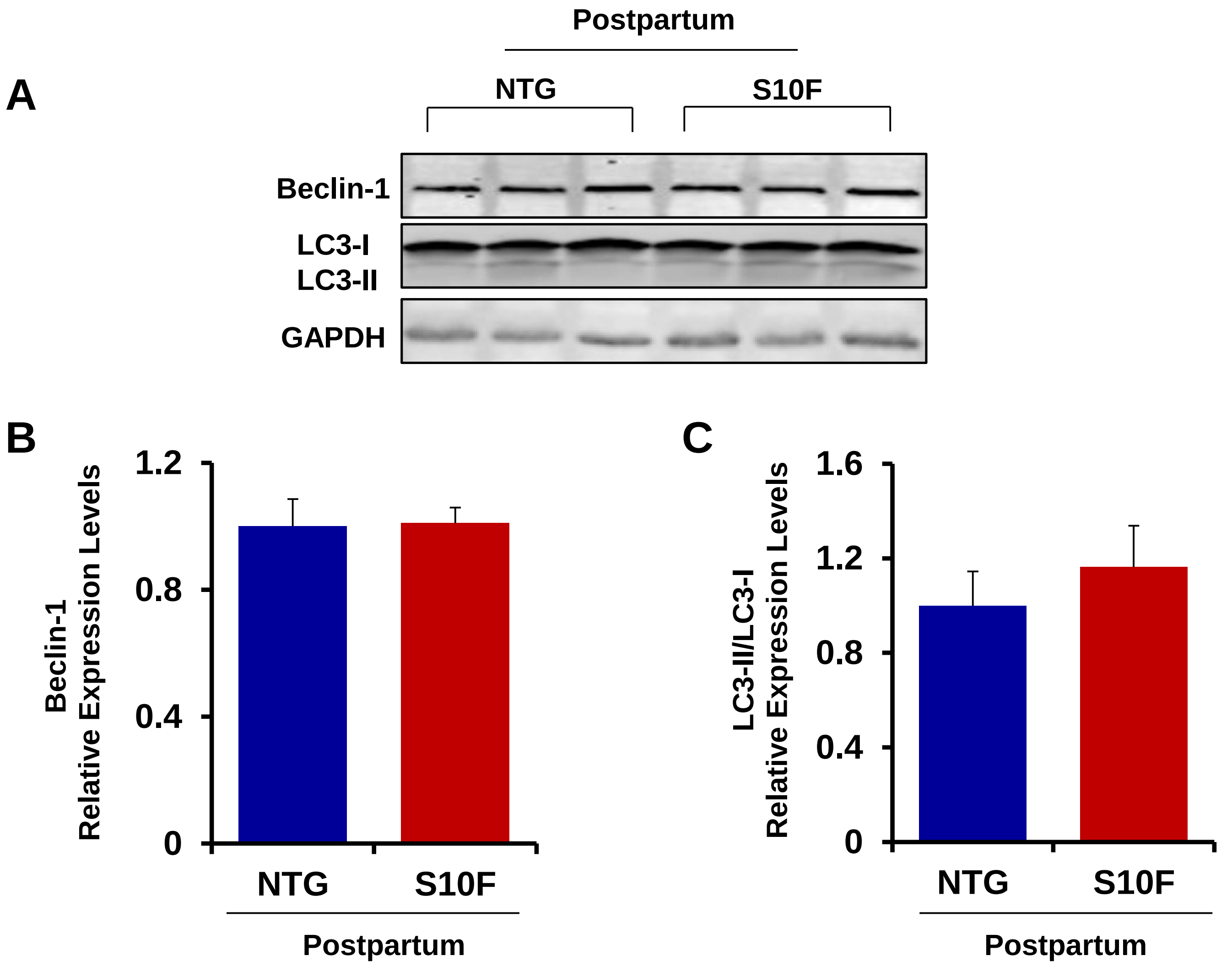

Supplementary Figure 2. Protein levels of Beclin-1 and LC3-II/LC3-I were unaltered following three pregnancies in S10F-Hsp20 hearts. (A) Representative western blots of Beclin-1 and LC3-II/LC3-I protein levels in NTG and S10F hearts following three pregnancies. (B) Quantitative analysis of Beclin-1 and LC3-II/LC3-I protein levels after normalization to the loading control, GAPDH. Values represent mean ± SEM; n=3 per group.
