# Supplementary material for: A novel human S10F‐Hsp20 mutation induces lethal peripartum cardiomyopathy
Source: J Cell Mol Med. 2018 May 15;22(8):3911–9. doi: 10.1111/jcmm.13665 (PMC6050507; doi:10.1111/jcmm.13665)
Supplement: Supplementary file 3 [file JCMM-22-3911-s003.pdf]

Table S1

Echocardiography Parameters in NTG and S10F-Hsp20 Female Mice

Group	EF	FS	LVEDV (µL)	LVESV (µL)	LVIDd (mm)	LVIDs (mm)	HR (beats/min)	SV (µL)	CO (mL/min)
**NTG**	57.8 ± 3.0	30.0 ± 2.0	63.3 ± 1.6	26.6 ± 1.3	3.8 ± 0.01	2.7 ± 0.05	376.7 ± 19.8	36.7 ± 2.7	13.9 ± 1.5
**S10F**	60.4 ± 1.6	31.9 ± 1.1	70.4 ± 3.6	27.9 ± 2.1	4.0 ±0.1	2.7 ± 0.1	402.2 ± 24.1	42.4 ± 2.1	14.0 ± 1.8

Supplementary Table 1. Echocardiography parameters in NTG and S10F-Hsp20 Female Mice at 6 months of age. EF indicates ejection fraction; FS, fractional shortening; LVEDV, left ventricular end-diastolic volume; LVESV, left ventricular end-systolic volume; LVIDd, left ventricular internal dimension at end-diastole; LVIDs, left ventricular internal dimension at end-systole; HR, heart rate; SV, stroke volume; CO, cardiac output. Values represent mean ± SEM; n= 3 for NTG and n=6 for S10F.
