# Supplementary material for: A novel human S10F‐Hsp20 mutation induces lethal peripartum cardiomyopathy
Source: J Cell Mol Med. 2018 May 15;22(8):3911–9. doi: 10.1111/jcmm.13665 (PMC6050507; doi:10.1111/jcmm.13665)
Supplement: Supplementary file 4 [file JCMM-22-3911-s004.pdf]

Additional Echocardiography Parameters in NTG and S10F-Hsp20 Mice Postpartum

Group	HR (beats/min)	SV (μL)	CO (mL/min)
**NTG**	430 ± 22.2	61.7 ± 3.9	26.6 ± 2.2
**S10F**	385 ± 5.5*	63.8 ± 7.4	24.5 ± 2.7

Supplementary Table 2. Additional Echocardiography parameters in NTG and S10F-Hsp20 Mice following 3 pregnancies. HR indicates heart rate; SV, stroke volume; CO, cardiac output. Values represent mean ± SEM; n= 5 for NTG and n=7 for S10F. *: $p<0.05$ vs NTG.
